# Implementing contingency management in family medicine: A qualitative inquiry on provider and patient preferences for a low magnitude reward program compatible with buprenorphine treatment

**DOI:** 10.21203/rs.3.rs-6347618/v1

**Published:** 2025-04-28

**Authors:** Samantha Ellis, Jax Witzig, Diego Basaldu, Brittany Rudd, Nicole Gastala, Alexandra R. Tabachnick, Sungha Kang, Tondalaya Henry, Nathan Stackhouse, Margaret Wardle

**Affiliations:** University of Illinois at Chicago; University of Illinois at Chicago; University of Illinois at Chicago; University of Illinois at Chicago; University of Illinois at Chicago; Northwestern University; Loyola University Chicago; University of Illinois at Chicago; University of Illinois at Chicago; University of Illinois at Chicago

**Keywords:** opioid use disorder, medication for opioid use disorder, contingency management, user-centered design

## Abstract

**Background:**

Contingency management (CM) is an effective yet underutilized behavioral intervention that uses rewards to improve outcomes in medication for opioid use disorder (MOUD) treatment. Prior implementation attempts have focused on specialized addiction clinics, using intensive daily treatment with methadone and high reward values (e.g. >$200 total). However, many people get MOUD from less specialized, more accessible, family medicine clinics. These clinics could also benefit from CM, yet present unique challenges for CM. Family medicine clinics typically use buprenorphine as their primary medication, which requires less intensive dosing schedules and thus provides fewer CM opportunities. They may also have lower institutional willingness to use high-value rewards. As an initial step in user-centered design of a low value reward (<$75 total) CM program for the family medicine context, we conducted qualitative interviews with patients and staff in the buprenorphine treatment program of a family medicine department. We gathered and analyzed qualitative data on CM knowledge, preferred program parameters, and implementation considerations.

**Method:**

Participants (*N* = 24) were buprenorphine treatment staff (*n* = 12) and patients (*n* = 12). Participants completed 30–50-minute semi-structured interviews, analyzed using rapid matrix analysis.

**Results:**

Participants had little experience with CM, but generally viewed CM as acceptable, appropriate, and feasible. Interviewees coalesced around having staff who were not providers with prescription privileges conduct CM, consistent rather than escalating payments, and physical rewards delivered in-person. Potential challenges included medical record integration, demands on staff time, and confirmation of patients’ goal completion.

**Conclusions:**

Patient and staff feedback was well-aligned, especially regarding rewards as an opportunity for staff-patient connection and the need for simplicity. Some consensus suggestions (e.g. non-escalating rewards) conflict with extant CM literature. Implications for implementation of CM in this setting are presented. These findings inform user-centered design and iteration of a CM program for this accessible, non-specialized family medicine setting.

## Background

The United States is now in the fourth wave of the opioid overdose epidemic, an ongoing public health emergency that has seen deaths continue to rise (Mattson, 2021) as well as widespread impacts on mental and physical health, housing, job stability, and community services like healthcare (Hagemeier, 2018). Medication for opioid use disorder (MOUD) is an empirically supported treatment that reduces risk of overdose (Wakeman et al., 2020) and improves mental and physical health (Amura, 2022). Methadone, a full opioid agonist, and buprenorphine, a partial opioid agonist, are the most common forms of MOUD (Wakeman et al., 2020). However, methadone can only be dispensed by specially licensed Opioid Treatment Programs (OTPs), and typically requires frequent clinic visits. Although rule alterations during the COVID-19 pandemic eliminated daily dispensing requirements, many providers still require frequent visits for methadone dispensing (Meyerson et al., 2024). In contrast, buprenorphine can be dispensed by any physician with an appropriate DEA registration, and generally is prescribed in a way that allows for fewer clinic visits – often weekly to monthly. Use of buprenorphine expands access by increasing provision of MOUD by non-specialist providers like family medicine doctors (Olfson et al., 2020), most notably in rural and underserved areas (Abraham et al., 2020). However, buprenorphine also has poorer retention than methadone (Bell et al., 2009; Degenhardt et al., 2023; Timko et al., 2016). Indeed, in a large sample of patients on Medicaid, most patients receiving buprenorphine discontinued the medication within 3 months, with around a third discontinuing in the first month of care (Samples et al., 2018).

Scientific studies aiming to improve retention in MOUD have found Contingency Management (CM) is an effective method (Bolívar et al., 2021). CM provides rewards (e.g., gift cards) when patients achieve treatment goals, such as attending visits or submitting opioid-negative urine samples. CM has been found to be effective across a number of targets, including abstinence, medication adherence, and attendance (DeFulio & Silverman, 2012; Dugosh et al., 2016; Griffith et al., 2000; Lussier et al., 2006; Prendergast et al., 2006). However, despite strong empirical support and the existence of high-quality materials for training MOUD staff in CM (Helseth et al., 2018), only 10% of front-line MOUD staff actually use CM (McGovern et al., 2004a). There have been prior attempts to bridge this implementation gap, but these have focused on specialized treatment settings. One was in the Veteran’s Affairs system, and took advantage of the VA’s integrated care model, different reimbursement structure, and on-site resources such as canteens to provide rewards (DePhilippis et al., 2018). Other implementations have been in partnership with registered OTP providers with a majority of patients receiving methadone (Becker et al., 2019a, 2021). Both the need to improve retention and the public health benefits of improving retention using CM may be greater in non-specialty settings prescribing primarily buprenorphine, yet there may also be greater challenges in these settings. Indeed, in two state-wide implementation efforts that included a variety of sites, the authors qualitatively observed that specialty or registered OTP providers appeared better able to implement CM than generalist settings (Parent et al., 2023). Despite this need, to our knowledge, there have not been intentional attempts to fit CM for less-specialized MOUD settings like family medicine.

Prior CM implementation work has highlighted barriers/concerns such as cost, staff perceptions that programs will not address the underlying problems in addiction, and implementation based on researchers, not practitioners (Carroll, 2014; Hartzler & Rabun, 2013; Kirby et al., 2006; McGovern et al., 2004b; Roll et al., 2009). Implementing CM in less specialized but more accessible primary care clinics that rely on buprenorphine may present additional challenges.

One is less frequent patient visits. The CM literature shows opportunities for reward should occur frequently (at least weekly if not more) for CM to be effective (Pfund et al., 2021); however, one advantage of buprenorphine is longer times between visits, which conflicts with this need. Indeed, this was one explanation for the greater success of OTPs in the state-wide implementation described above: more intensive treatment schedules provide more opportunities for CM (Parent et al., 2023). Another challenge is constraints on total reward value. The CM literature shows that rewards must meet a certain threshold to be effective (Becker, DiClemente-Bosco, Rash, et al., 2023). Consistent with this, prior implementations of CM utilized total reward amounts over $200 (Becker, DiClemente-Bosco, Scott, et al., 2023; Becker et al., 2019b; Hartzler et al., 2014; Petry et al., 2012a, b). However, the Centers for Medicare and Medicaid Services (CMS) sets a $75 per year per patient limit on rewards/incentives to patients, with larger amounts considered illegal “kickbacks” (Clark & Davis, 2023). The Office of the Inspector General (OIG) has held that this does not prohibit carefully-run higher-value CM programs, and has issued favorable rulings to some specific higher-value CM programs (Clark & Davis, 2023). However, large generalist health care organizations that do not specialize in opioid treatment may be more comfortable staying within the “safe harbor” limit of the OIG rather than seeking a ruling. This would limit CM programs to low-value rewards (Gerra et al., 2006; Levine et al., 2015; Marcovitz et al., 2016; Noe & Keller, 2020).

Although specific barriers and facilitators may vary in a family medicine setting, we believe the same implementation techniques that have been used to adapt CM to more specialized settings can be used effectively here (Becker et al., 2019b, 2021). These include first assessing the organization’s viewpoints on and capacity for CM, using user-centered design in implementation (Lyon & Koerner, 2016), developing detailed contingency plans, and continued training and partnership (Becker, DiClemente-Bosco, Rash, et al., 2023). Here we report on the first step in this process: conducting semi-structured qualitative interviews of patients and providers to inform the user-centered design of a CM program for a family medicine clinic embedded within a federally qualified healthcare center (FQHC). These results will inform development of a CM program that improves outcomes and is primed for adoption and implementation in non-specialized clinics. User-centered design has been identified as critical to the uptake and adoption of CM, but to our knowledge this is the first that the principles of user-centered design have been used to identify considerations for implementing CM in a non-specialized primary care setting. This work also extends prior examples of user-centered design of CM (Becker et al., 2019b) by including patient perspectives in addition to staff.

We first engaged in pre-design work with key decision-makers in our community partner clinic (the senior MOUD provider and program coordinator), to identify program elements that were constrained by either scientific findings or clinic needs and thus would not be open to design input. First, the initial month of treatment was the only time patients would be seen often enough (at least weekly) for effective CM per the literature, so the program would enroll patients for their first month of treatment only. This is a comparatively short time period, especially given findings that CM effectiveness improves with longer treatment times (Petry et al., 2018; Roll et al., 2013). However, it at least covered the period with the most treatment drop-out, both per national findings and the observation of local program staff. Further, recent meta-analyses show long-term benefits even after CM discontinuation (Ginley et al., 2021). Although this was speculative, it seemed possible that better adherence and improved alliance with staff in the first month could provide some lasting benefits. Second, our partner clinic has a patient-centered, harm-reduction approach, not requiring abstinence as a goal. Thus, two non-abstinence behaviors were selected as targets: weekly attendance and weekly personal goals. Personal goals were strongly desired by clinic decision-makers, consistent with their patient-centered approach. However, another important principle of CM is that target behaviors should be clear and objectively verifiable. Personal goals are more difficult to verify, and, although they have been used previously (Lewis & Petry, 2005) they are less common targets of CM. Thus, we decided to simultaneously design two parallel programs – one targeting personal goals and one targeting attendance – to increase our odds of ultimately delivering an effective intervention. Third, to address decision-maker concerns we stayed within the <$75 “safe harbor” amount for incentives suggested by the OIG. As noted above, the literature suggests that $75 per year may not be sufficient for effective CM; however, many of the studies this statement is based on used abstinence as a goal. “Easier” behaviors, such as attendance, may be influenceable using smaller rewards, as demonstrated in prior studies (Hartzler et al., 2023; Kropp et al., 2017). Thus, this constraint also confirmed our choice of non-abstinence goals. Finally, decision-makers agreed that it would be important to use the existing electronic medical record system (EMR) (i.e. Epic - Epic Systems Corporation) to track goals and rewards, rather than custom spreadsheets or other methods used in prior CM implementations (Becker, DiClemente-Bosco, Scott, et al., 2023).

With these broad guidelines established, we then conducted semi-structured interviews with clinic patients and staff to systematically collect end-user input on implementation factors and remaining key design features, with the overall goal of informing an acceptable, adoptable, and feasible CM program for this context. We used rapid matrix analysis, a cutting-edge qualitative technique, to provide fast feedback for design and implementation (Averill, 2002).

## Methods

### Recruitment and participants.

Recruitment occurred at a family medicine clinic at a federally qualified health center in Illinois. This FQHC is affiliated with a larger academic medical system but is a public-community partnership operated separately from the academic medical center’s general outpatient clinics. The FQHC predominantly serves patients from structurally marginalized communities, many of whom live at or below the Federal Poverty Level, have public insurance, or are uninsured, and sees all patients regardless of their ability to pay. For this study, eligible patients had to be over 18 years old, in their first 3 months of buprenorphine treatment for OUD in the clinic, and able to read and speak English at an 8^th^ grade level to complete written informed consent. Eligible staff had to have been involved in MOUD patient care for at least 3 months and able to read and speak English at an 8^th^ grade level to complete written informed consent. We interviewed 12 clinic staff and 12 patients for a total of 24 participants. Table 1 presents the demographics of the final sample of both staff and patients.

### Procedures.

All procedures were approved by the Institutional Review Board at the University of Illinois Chicago. Patient participants were initially identified via review of medical records, then either approached by study staff during regular clinic visits or given a referral card by clinic staff containing a basic description of the study and contact information. Staff participants were recruited via e-mail, visits to clinical meetings by study staff, and referrals by other staff and program leaders. We purposely and successfully recruited across the full range of staff positions with clinical contact with MOUD patients, including physicians/residents, Advanced Practice Nurses (APNs), other nursing staff, program coordinators, clinical psychologists, social workers, and social work aides. We also asked all interviewees who in the program (either patients or staff) we should speak with and attempted to recruit any people named. Interested participants completed a brief screening questionnaire in-person, over the phone, or online. Eligible participants were scheduled for a one-hour combined consent and interview session, in-person or via secure video conferencing. Written informed consent was obtained using either paper consent form or an online REDCap form (Harris et al., 2009, 2019).

### Qualitative Interviews.

The qualitative interviews were 30–50-minute semi-structured interviews conducted by one of six trained research assistants (all at least Master’s level with prior interviewing training), supervised by a licensed clinical psychologist (MW). First, the interviewer briefly described the concept of CM. The following semi-structured interview questions probed general knowledge/prior experience with CM, perceptions of acceptability, appropriateness, and feasibility of CM, including barriers and facilitators, and specific design features including preferred reward types and schedules and how to track patient goals. We also addressed some logistics with staff only, including what positions were best suited to executing the program, how the program would fit into the clinical workflow, and use of the EMR system to track goals and rewards. See Supplemental Material 1 for full interview scripts.

### Summary Templates.

Data from the qualitative interviews were compiled for matrix analysis via summary templates. Summary templates are an established means of collecting and organizing qualitative data before analysis and interpretation (Hamilton, 2013), and can reduce costs and time compared to traditional interview transcription (Hamilton, 2013; Nevedal et al., 2021). Recent research also suggests that summary templates are particularly useful for quickly extracting information about new interventions in public health (Gale et al., 2019; Taylor et al., 2018). Thus, summary templates were deemed an excellent fit for the present study.

The summary templates were developed by a research team member with prior experience developing and analyzing similar templates (JW). Rather than transcribe the interview verbatim as in traditional transcription, one of four trained analysts summarized the interviewee’s responses into succinct, clear insights that could be easily reviewed (Harkness et al., 2022; Renfro et al., 2022). See Supplemental Material 2 for finalized summary templates for patients and staff and Supplemental Material 3 for further discussion of summary template procedure.

### Matrix analysis.

Once summary notes were taken, the five-member analyst team began the matrix analysis. Matrix analysis is an increasingly popular strategy for rapidly analyzing and interpreting qualitative or mixed-methods data (Averill, 2002). Matrix analysis begins by creating a grid, wherein rows are unique observations (e.g., individual interviews) and the columns are interview guide questions or domains (Averill, 2002; Hamilton, 2013). Our team created one grid for patient interview data and another for staff interview data.

Once the information was pasted into the matrix grids, each grid was separately reviewed by the full analyst team to identify themes and patterns. There is no one universally accepted way to complete this process; however, our team followed the recommendations of Uscher-Pines et al. (2020) and analyzed the matrix grid data by *quantifying recurrent themes* and *identifying emphatic themes* as further discussed in Supplemental Material 3.

#### Reporting of results.

Once thematic quantification and identification of themes were complete, results were compiled in tables denoting the recurrent themes and their corresponding final tabulations, as well as a separate column denoting emphatic themes. These tables were used as the basis for reporting the results.

## Results

### Knowledge of Contingency Management

We first assessed prior knowledge of and experience with CM.Of the 12 patients, only one reported an experience with CM.There was more experience with CM within the staff, with 4 of 12 reporting experience with a CM program – in other clinics as well as part of a research study. Of the 4, one reported experience in the Veterans Association implementation of CM (DePhilippis et al., 2018).

### Implementation Constructs

To elicit information about the perceived acceptability, appropriateness, and feasibility of CM in this context, along with common concerns about CM (e.g. that people might misuse CM rewards to buy drugs) (Scott et al., 2020), we next asked participants about the perceived helpfulness of a rewards program, how comfortable they would be with a rewards program at the clinic, and the perceived practicality of a rewards program in the clinic, including perceived barriers and facilitators (Table 2). Acceptability, appropriateness, and feasibility are multifactorial and interrelated, and thus we do not map them 1:1 onto these specific items; rather we expected these items to elicit themes relevant to these constructs. Results of the specific questions are presented in Table 2. 75% of patients and 92% of staff stated that a CM program would be helpful, although patients (but not staff) expressed some concerns that people might use the rewards inappropriately (e.g., using it to buy heroin). 100% of patients and 83% of staff expressed feeling comfortable with a rewards program in the clinic, with patients citing positive views of the clinic as a reason they thought such a program would be appropriately run there, and staff generally approving while also noting the need for clear ground rules. Views on practicality were more split, with patients overwhelmingly viewing CM as practical and the staff having mixed responses though majority affirmative. Patients noted few barriers or facilitators aside from questions about funding sources, while staff described overwhelm as a potential barrier, suggesting that having a designated “point person” for the program would be one of the most important elements for practicality.

Following recurrent theme identification in these interview guide questions, themes across all three questions were mapped onto the implementation constructs of acceptability, appropriateness, and feasibility by trained raters to identify implementation areas of most concern. In this analysis, two reviewers independently coded themes across patients and staff into categories and then reached consensus. This additional analysis established recurrence (two or more mentions) of themes within implementation constructs. Acceptability had the fewest mentions (2), via themes of “staff feel overall support for the program” and “positive comments about program.” For appropriateness (6), themes of usefulness and helpfulness of the program aligned, as well as noted concerns. Feasibility saw the highest number of coded themes (8), with clear discussion of “need (for) boundaries/rules,” potential for overwhelm (staff and patient), and multiple themes regarding needing a clear plan (e.g., point-person, training, buy-in).

### Program Specifics

We next asked questions about program specifics, such as how to split the $75 total, possible personal goals and confirmations, and what kinds of rewards patients might like (Table 3).Given the $75 limit,patients and staff were split on whether rewards should be distributed evenly (e.g., three payments of $25) or unevenly (e.g., $50 then $25) across weeks. However, they most frequently identified 3 payments of $25 as preferable. The need to wait for rewards and practice delayed gratification was also brought up, primarily by patients – and included suggestions such as not getting rewards for the first week. Additionally, staff were highly likely to emphasize that rewards should be contingent on patients’ adherence to the program, though patients should be “given grace” if they were non-adherent. An emphatic theme that arose from this domain was that staff felt that patients should have more input in designing the program than staff should.

Patients noted a broad range of potential goals; thus, only goals that were mentioned by at least four interviewees (1/3 of the sample) in either group are noted in Table 3. Additionally, an emphatic theme for this domain was the goal of finding a sponsor. Staff also noted a wide array of goals but focused more heavily on treatment; although only three staff interviewees mentioned interpersonal goals (e.g., repairing relationships), this goal was also an emphatic theme – suggesting that it is important to attend to.

Patients largely felt that there should be physical proof involved in confirming goals. Staff largely felt that patient-provider check-ins would be an acceptable means of confirming patient goals. Confirming goals via check-ins arose as especially important given this theme was both recurrent and emphatic. Staff also felt that hard documents (e.g., meeting sign-in sheets) would be appropriate. One-quarter of the sample mentioned self-reporting as a means of confirmation. An emphatic theme that arose for this question was that staff strongly felt the program should involve informed consent, which would ensure that patients understand their involvement, the available rewards, the rewards schedule, etc.

Patients noted a wide range of rewards that would be appropriate; thus, only rewards mentioned by four or more interviewees (1/3 of the sample) are noted. Emphatic themes for this domain included transit support (given that this was both a recurrent and emphatic theme, it may be especially important) and basic necessities (e.g., toothpaste).Staff endorsed a smaller range of ideas than patients. An emphatic theme that arose for this domain was that staff felt the reward options should be “patient-led” -- i.e., that patients should decide what rewards are available.

For giving rewards, overwhelmingly, patients and staff felt that they should be given in person. In fact, the only other method patients mentioned (mail) was only spoken about in a negative sense given that by mail, rewards may be lost, there is no way to confirm the rewards’ receipt, and patients may not have reliable home addresses.Participants were split on when the CM process should happen within an appointment; some stated that it should be part of the appointment with the provider itself, while others said it should happen directly before or after.

### Staff-Only Logistics.

We then covered some additional logistics questions with staff only (Table 4), includingthe best staff for program roles (explaining the program, giving rewards), fit into workflow, and use of medical records. Generally, interviewees recommended that support staff explain, confirm progress, and give rewards (versus “providers” – a category for this clinic which includes physicians, APNs and others with a specific designation for billing services and prescribing medication). Regardless of who is ultimately selected for this role, interviewees felt that the person should have subject matter expertise with addiction.

In consideration of workflow fit, staff highlighted a need for designated staff to support the program (particularly social workers or social worker aides), that a new position would need to be created to manage the program, and that administrative staff could also support the program (very little information was given as to the last point). A quarter of participants also noted that using features of the medical record would be helpful for tracking purposes (such as chat or “dot phrases”), though this was discussed much more extensively in questions specific to the use of EMRs.Above all, interviewees felt that the program would require clear communication regarding rollout, workflow integration, and other logistics with buy-in from staff. Surprisingly, participants identified few areas where the CM program could cause problems for the clinic’s workflow.

In response to specific questions about the EMR, most participants felt that existing tools could and should be leveraged in service of the CM program. Despite these positives, interviewees also noted that there were some anticipated difficulties with using the EMR (lack of expertise, effort). We also asked specifically about the Epic Care Companion, which is an extension of Epic MyChart, the patient portal allowing for communication between provider and patient, specific “assignments” or measures to be given to patients, and overall greater patient involvement in their care. When asked, eight of the 12 interviewees felt it would be a good idea to use a Care Companion module for the contingency management program. However, relatively few individuals were familiar with the functionality of the Care Companion.

## Discussion

This qualitative analysis solicited viewpoints on CM and its implementation as part of buprenorphine treatment of OUD from patients and staff of a family medicine clinic in the first step of user-centered design of CM for the same clinic. We solicited knowledge and perspectives on CM, how to divide the $75 total reward, preferred types of rewards, how to give rewards, potential patient goals, and staff-only logistics (e.g., what positions are best suited to being part of the program, workflow, using Epic) to inform program design. Most patients had no experience with CM; however, they broadly viewed such a program as helpful, were comfortable with the clinic offering such a program, and reported it as practical for the clinic. More staff had experience with CM, although still a minority, and staff as well had an overall positive viewpoint (helpful, comfortable, practical). Overall, the data presented suggests support for the implementation of CM from patients and buprenorphine treatment staff. Both patients and staff provided specific considerations for implementation, some of which aligned and some of which contrasted, both across groups and with the larger literature on CM.

Best practices in contingency management have been established regarding target behavior, incentive (choice, magnitude, frequency), and duration of the intervention (Rash et al., 2019). As noted in the introduction, scientific and logistical constraints dictated some of these for our program (e.g. weekly reward, amount of reward), and we did not further query participants on these pre-determined points. However, within these limitations, we did ask patients and staff how they thought a CM/rewards program should be designed without presenting “best practices” to them. As might be expected, we identified areas where our participants spontaneously agreed with best practices, as well as differed from them. These results indicate areas where researchers may need to conduct more education to build staff and patient “buy-in,” invest more resources in the implementation process, or even consider compromising to produce feasible programs. First, the literature states target behaviors should be observable and measurable, and have patient buy-in (Petry, 2000). Staff agreed on the idea of patient buy-in, but staff more often suggested using self-report for verification of target behaviors – whereas objective verification is thought to be a critical component of CM (Petry, 2000). Regarding incentives, staff and patients approved of standard incentives including both prizes and cash-equivalents (gift cards; Kropp et al., 2017). However, escalating and resetting reward amounts are recommended to produce longer durations of behavior in high reward programs(Petry, 2000). Although uneven/escalating amounts were mentioned, our participants generally favored the ease of splitting the amount equally across weeks, perhaps indicative that escalating payments is less generalizable to low reward programs. CM best practices also suggest random draws as an effective method to lower the cost of rewards (Petry et al., 2006), which was not mentioned by our participants. The literature indicates incentives should be given immediately after demonstration of the target behavior (Washio et al., 2011). Our participants generally agreed incentives should occur right before, during, or after the visit (i.e., as immediately as possible), and highly preferred in-person delivery, in line with these principles. However, patients also mentioned a need to promote delay of gratification that directly opposes best practices, and conflicts with research findings that delay of gratification is impaired in those with substance use disorders (Petry, 2000). Overall, the key areas for implementation researchers to consider additional education and support were around the need for objective verification (primarily with staff in our sample), the logistics of escalating or random draw reward schedules, and the benefits of reward immediacy (primarily with patients in our sample).

We also saw areas of overlap and difference with prior investigations of staff and patient attitudes about CM and other projects conducting user-centered design of CM. Although it is important to note this was not intended to be a representative sample, we did not see the staff concerns about the misuse of rewards or belief-based objections to CM in patients that have been noted in other work (Kirby et al., 2006; Oluwoye et al., 2023; Rash et al., 2012). Future representative studies will be needed to see if this indicates fundamentally different attitudes in family medicine MOUD staff and patients, shifts in attitudes over time, or was unique to our setting and sample. We also had different findings to one prior user-centered design of CM that interviewed leaders and frontline counselors in OTPs (Becker et al., 2019b). We found a similar low familiarity with CM, but while OTP respondents preferred “higher-level” positions (e.g., program director) to run CM and administer rewards, our staff interviewees favored “lower level” positions (e.g., social work technicians). This might reflect the less specialized nature of our setting, where people in “higher level” positions may not be particularly involved in addiction care.

The presented study has several limitations. First, as noted above, this is not a representative survey of staff or patients in family medicine treatment settings, so our findings must be considered specific to our participants and setting. They may provide directions for inquiry in larger studies but are not generalizable. We also saw a low level of familiarity with CM, which may have limited the ability of participants to respond meaningfully to our questions. Greater familiarity with CM could have generated additional novel ideas about CM program specifics, including facilitators and barriers. Finally, we also must consider the representativeness of the sample, which was primarily determined by who agreed to an interview. Our patient respondents were slightly more likely to be female and were about evenly split between people identifying as White or Black, with only one Hispanic-identifying respondent. The demographics of the MOUD program are unknown; however, the clinic patient population in general is 39.2% male, 53.7% Non-Hispanic Black/African American, 28.6% Hispanic, 9.9% Non-Hispanic White, 2.8% Non-Hispanic Asian and 3.5% Non-Hispanic other race. That suggests under-sampling of male and Hispanic patients (the latter possibly due to language constraints, as we had only English-language materials). Further, although we were careful and successful in recruiting across the range of staff positions with clinical contact with MOUD patients, the staff respondents were majority graduate-level educated Black women. Although exact demographics for the clinic staff are not known, this at least strongly suggests under-sampling of male and White staff. Therefore, in future attempts, it will be important to first determine program-specific demographics and aim to have a sample as true to the program patients and staff as possible, particularly attending to recruitment of male respondents and to having materials across the languages spoken by patients.

Despite these limitations, this study represents a novel attempt to conduct qualitative interviews to inform user-centered design of a CM program for use in conjunction with MOUD in a family medicine setting. Our results suggest that though there are logistical differences with implementing CM in a non-specialized setting vs an OTP, patients and staff are receptive and willing to participate in the adaptation of CM for long-standing program success. Next steps in this specific program of work are to use the information gathered to guide the design of final CM programs in collaboration with a community design board including patients, staff and administrators, and then to test the impact of those programs on metrics important to the clinic, including attendance/no-show rates, medication adherence and abstinence. Ideally, this will serve as a pilot study for a future large-scale stepped-wedge design implementing this program across family medicine MOUD settings. We hope this will also serve as a guide for other work that aims to further increase availability of CM by using user-centered design to adapt this evidence-based intervention to specific settings in need of this powerful therapeutic tool.

## Data Availability

The datasets used and/or analyzed during the current study are available from the corresponding author on reasonable request.

## Abbreviations

MOUD: Medication for opioid use disorder
OTPs: Opioid Treatment Programs
CM: Contingency Management
CMS: Centers for Medicare and Medicaid Services
OIG: Office of the Inspector General
FQHC: Federally qualified healthcare center
EMR: Electronic medical record system
APNs: Advanced Practice Nurses
